# Treatment of Gonorrhœa

**Published:** 1846-10

**Authors:** 


13. *Treatment of Gonorrhœa.*—Mr. McDonald, (Lancet,) recommends the following treatment in this affection. Smear a bougie with ointment of the nitrate of silver, (℞. Argent Nitratis ʒj; adipis ℥j;) introduce it into the urethra for about three inches, and allow it to remain two or three minutes. Two or three applications have been found to cure the disease; and if used in the acute stage, one application is generally sufficient.—*Ibid* in *Ibid.*

